# Mass extinctions, their causes and consequences: an interview with Douglas H. Erwin and Shuzhong Shen

**DOI:** 10.1093/nsr/nwad250

**Published:** 2023-09-22

**Authors:** Shucheng Xie

**Affiliations:** State Key Laboratory of Biogeology and Environmental Geology, China University of Geosciences at Wuhan

## Abstract

‘Mass extinctions’ have been a hot topic for several decades. What triggers a mass extinction? How does a mass extinction impact the evolution of life? How does our ecosystem recover after a mass extinction? These questions attracted the interest of both scientists and the public alike. NSR spoke to two renowned researchers in the field of mass extinctions: Prof. Douglas H. Erwin from the National Museum of Natural History of the USA, and Prof. Shuzhong Shen from Nanjing University, China. Prof. Erwin has been interested in the end-Permian mass extinction since graduate school. He has worked on a variety of problems, from Permian gastropod systematics to the origin of animals. Currently his work focuses on the nature of evolutionary novelty and innovation. Prof. Shen's research career is centered upon the end-Permian mass extinction, Permian stratigraphy and global correlations, with taxonomic expertise on brachiopods and conodonts. The International Commission on Stratigraphy recognizes his outstanding singular contribution to stratigraphy and awarded him the ICS Stratigraphy Medal in 2019.

**NSR:** ‘Mass extinctions’ have been a hot topic for several decades among scientists and the public. So, what is a mass extinction, and how severe was it in Earth history?

**DHE and SZS:** Interest in mass extinctions has greatly increased since 1980 when the Cretaceous-Tertiary (K-T) mass extinction (∼66 million years ago – Ma) was hypothesized to have been triggered by the impact of an extra-terrestrial object [1]. This hypothesis grew from the discovery of abundant iridium in a thin layer at the K-T boundary near Gubbio, Italy and many other sections from around the world. At almost the same time, Jack Sepkoski compiled a database of the first and last occurrences of the Phanerozoic marine families [2] (later expanded to genera). Working with David Raup, Jack used the generic data to demonstrate an apparent 26-million-year periodicity of mass extinctions from the end-Permian (251 Ma) to today (a pattern that has been widely discussed). But massive biotic crises have been recognized since Cuvier and Phillips in the 19th Century, and many of them provide the structure of the Geological Time Scale. Since Norman Newell's work in the early 1960s mass extinctions have been recognized as global losses above background levels of extinction in taxa across many different clades. After Raup and Sepkoski's work in the 1980s, our understanding of mass extinctions in the geological past has expanded enormously, with detailed studies of each event, as well as synoptic studies of biodiversity both on land and in the sea. Many biotic crises have been identified over the past 600 million years. Of the canonical five great mass extinctions: end-Ordovician, late Devonian, end-Permian, end-Triassic, and end-Cretaceous [3], questions have been raised about whether the late Devonian and end-Triassic extinctions both rank at the same level as the other three. The late Devonian episode seems to have involved a series of biotic crises and experienced a long diversity decline from early Middle Devonian to the end-Devonian rather than a single crisis around the Frasnian/Famennian boundary, and some paleontologists have also argued that the end-Triassic differed as well.

**Figure 1:**
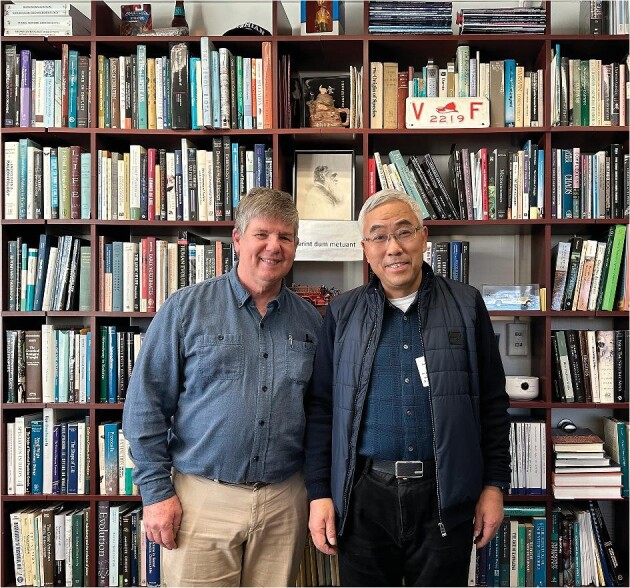
Profs. Douglas H. Erwin and Shuzhong Shen at Erwin’s office in the National Museum of Natural History, a part of the Smithsonian Institution. This photo is the first time they could meet since the pandemic. The photo between Erwin and Shen is of Charles Darwin, and above that is a teapot made to celebrate the designation of the Meishan Section as the Global Stratotype Section and Point for the Permo-Triassic boundary. The lid of the teapot (barely visible) is a reproduction of the conodont *Hindeodus parvus. (Courtesy of Shuzhong Shen)*

An essential contribution to understanding these events, often downplayed in some western countries, is the fundamental work of describing fossils and developing the precise

Such integrated studies are very time-consuming and expensive, but they are essential to understanding the causes and dynamics of extinction and recovery.

—Douglas H. Erwin and Shuzhong Shen

biostratigraphic and chronostratigraphic frameworks that allow geologists to correlate the same time horizon across a region or globally. Some younger geologists do not realize that high-resolution global correlations were rarely possible before the late 1950s. This kind of work may not produce high-profile publications, but it is an essential scaffold to eventually generating such papers.

There are some key points about mass extinctions that frequently get lost. First, mass extinctions were initially recognized in the record of marine fossils, primarily marine invertebrates, and some microfossil groups. Consequently, we can only identify mass extinctions over the past 540 million years, although there is a current debate over the possibility that biotic crises also occurred in the mid- and late Ediacaran, just before the Cambrian animal radiation. Second, the marine fossil record is dominated by species that lived in shallow marine environments, were geographically widespread, numerically abundant, and sufficiently durably skeletonized to be preserved as fossils. As all paleontologists are aware, this means that we have much less understanding of the dynamics of species in the deep sea or open ocean. Third, because the marine fossil record is generally much richer than that of plants, insects or vertebrates on land, paleontologists have tended to recognize past biotic crises in the marine fossil record, and then ask what happened on land. Indeed, our US-Sino joint research group recently spent a few years studying many sections in South China, examining the relationship between extinctions on land and in the sea during the Permian-Triassic transition. By integrating fossil data, sedimentological evidence, geochemistry and high-resolution age-dating, this research has demonstrated a gradient in extinction with latitude and through time, with much of the terrestrial extinction after the extinction in the oceans. Such integrated studies are very time-consuming and expensive, but they are essential to understanding the causes and dynamics of extinction and recovery. Building such datasets will increasingly permit us to study the internal dynamics of extinction processes, as discussed further below.

**NSR:** What are the causes and triggers of past mass extinctions? Are all mass extinctions catastrophic? And what are the consequences impacting on the evolution of Earth life?

**DHE:** When I taught history of life on Earth in the late 1980s, scientists had proposed more than 30 different causes for the end-Cretaceous mass extinction, from global warming and cooling to a suggestion that the spread of flowering plants had led to widespread allergies among dinosaurs (I am not making that up!). These pre-dated the discovery of the spike of iridium marking an impact at the Cretaceous/Paleogene boundary, a discovery which dramatically altered research on this event. In my first book on the end-Permian mass extinction [4], there was an equally long list of potential causes, from biotic and provincial homogenization from the formation of the supercontinent of Pangaea, nutrient depletion, bombardment of cosmic radiation from a nearby supernova, or increased salinity to volcanism, global cooling, and anoxic waters.

**DHE and SZS:** There were three reasons why so many different hypotheses thrived. First, the Permian-Triassic boundary sections are scarce in most parts of the world except China and adjacent regions, and before the 1970s and 1980s western geologists knew little about the discoveries of Chinese geologists. Most western geologists believed that a major drop in sea level occurred at this time, and many linked the extinction to global regression. Chinese geologists and paleontologists had a far better understanding of this event than anyone else in the world, but it was many years before this wealth of knowledge was appreciated outside China. Second, there were few constraints on the pace of the extinction event. Did the extinction take 10 million years, from the late Guadalupian through the end of the Permian, as Sepkoski suggested in the early 1980s, 1 million years, or just a few hundred thousand years? In the absence of an independently established temporal framework it was difficult to test and evaluate different hypotheses. Finally, and rather bluntly, there was just a lot of shoddy thinking. Many commentators on mass extinctions had rather jumbled views of causality and failed to distinguish the proximal causes of extinction (which might be anoxia or increased temperatures) from the ultimate, triggering causes.

Thirty years later detailed studies of many sections including detailed fossil analyses integrating with geochemical treatments and well-developed high-precision geochronology have made testing alternative hypotheses far easier than before. This has not eliminated controversy, of course [5]. But we hope that today's hypotheses have firmer empirical bases. This is true both for the end-Permian events we have studied as well as the other mass extinction episodes.

As mass extinctions have been better studied, it has become apparent that each of the well-known events was very rapid, occurring over timescales of tens of thousands of years rather than millions of years. Such a temporal framework sharpens our focus on causes. The only impact associated with a mass extinction is at the end of the Cretaceous, while massive flood basalts and other extreme volcanism are associated with the end-Guadalupian, end-Permian, end-Triassic and end-Cretaceous, which probably led to associated changes in climate. Today the important research questions really involve connecting these ultimate causes, such as volcanism, with the direct killers that caused species and

…we hope that today's hypotheses have firmer empirical bases.

—Douglas H. Erwin and Shuzhong Shen

ecosystems to disappear. As discussed further below, this is a promising but very challenging research frontier. If each of these events is equally rapid but resulted from different triggers, this may provide an important clue about the resilience of ecosystems. It is possible that in each case environmental changes pushed ecosystems to a tipping point where they began to collapse, and there may be greater similarities in the patterns of collapse than in the initial causes.

The evolutionary consequences of these mass extinction episodes on the history of life remain one of the most fundamental questions. English geologist John Phillips recognized the Paleozoic, Mesozoic, and Cenozoic eras in the 1840s because of the fundamental discontinuities the end-Permian and end-Cretaceous mass extinctions imposed on the history of life. This remains true today, although our views are far more nuanced. For example, many detailed studies have examined the dynamics of angiosperms, placental mammals, birds, and other groups from the late Cretaceous into the Cenozoic, evaluating how the diversity, morphology and functional ecology of each group responded to the mass extinction. Similar studies have been published for some of the other events (although more are needed). There remains, however, an ongoing debate between paleontologists who see the mass extinction episodes as fundamental transitions in the history of life and others who view them as temporary interruptions to longer-term evolutionary trends. There is some truth to each perspective, depending on the evolutionary question being addressed. Mass extinctions have certainly provided new ecological and evolutionary opportunities for many clades, both in the sea and on land.

**NSR:** There are many remaining puzzles concerning mass extinction, so what remains unknown, and what needs more investigation in the near future?

**DHE and SZS:** Despite decades of research on the major extinction episodes, there is still much to be learned about their details, and no lack of controversy. Although the end-Cretaceous and end-Permian events are better studied than other episodes, many open questions still remain, and new techniques are sure to provide new insights into these two events. Here we highlight three areas that warrant investigation in the near future: the internal dynamics of extinction episodes, patterns of post-extinction recovery and the dynamics of other biotic crises.

One of the most perverse results of our high-resolution dating of the end-Permian mass extinction over the past several decades with our late colleague Sam Bowring of MIT, is that each time geochronologic dating improves, we find the extinction event was even more rapid than we can resolve. Our group has shown that the extinction occurred in less than 60 000 years and perhaps even less than 31 000 years [6]. Other studies have found similar rapidity for the end-Devonian and end-Cretaceous events. Coupled with other high-resolution chronologic approaches such as astronomical cyclicity, we have the prospect of identifying early warning signs of mass extinctions (although this may require abundance data and not just taxonomic diversity data) and the possibility of peering ‘inside’ extinction episodes to unravel their internal dynamics. As

Here we highlight three areas that warrant investigation in the near future: the internal dynamics of extinction episodes, patterns of post-extinction recovery and the dynamics of other biotic crises.

—Douglas H. Erwin and Shuzhong Shen

paleontologists we have largely been limited to comparing species and clades that became extinct with those that survived as we try and divine extinction causes. But with sufficient high-resolution data we may be able to examine in some detail the progress of biotic collapse. How do ecological networks unravel? Is the loss of primary productivity the first sign of a mass extinction? What is the biogeographic pattern of biodiversity loss? Several recent studies have compared the progress of extinction on land and in the oceans, and further advances may illuminate the relative pacing of extinctions in these two realms. Expanding extinction studies beyond diversity to incorporate changes in phylogenetic structure, morphologic disparity, and functional diversity, as several recent studies have done, is another promising avenue for future work.

The biotic expansions following mass extinctions has hardly been ignored over the past few decades, with several important papers covering diversity, ecology, and geochemical cycles. Nonetheless, this remains a promising area for field and empirical work, and for modeling of diversity and ecological dynamics. The improved stratigraphic and temporal resolution described in the previous paragraph applies to post-extinction intervals as well, and research has already shown that biotic recoveries contain interesting dynamics. Why do some clades survive a biotic crisis yet disappear during the recovery interval? What are the relationships between increases in taxonomic or phylogenetic diversity, functional diversity, and ecological processes? When during a recovery do new morphologic novelties or new clades appear in the fossil record? Is there a latitudinal or geographic structure to biotic recoveries? These and other questions will fuel ongoing research in this area.

There is much to be learned about the end-Ordovician, late Devonian, Guadalupian and end-Triassic mass extinction episodes, as well as smaller biotic crises. How do the rates, selectivity, dynamics and causes of these events compare to other events? If all biotic crises were equally rapid, for example, this might indicate similar dynamics of biotic collapse, independent of the triggering causes. Do smaller biotic crises have similar evolutionary consequences to events such as the end-Permian mass extinction (EPME), interrupting ongoing evolutionary patterns? Or are they more similar to non-extinction intervals? There appear to have been several very extensive biotic crises during the Cambrian (and at least one in the Ediacaran Period), but these have received less attention than Ordovician and younger biotic crises. They certainly deserve more detailed study. Although more recent biotic crises such as the Paleocene-Eocene Thermal Maximum (PETM) are often considered the most useful analogues to the ongoing biodiversity crisis, in part because of the rapid global warming associated with the PETM, the earlier biotic crises may provide critical insights, particularly if common patterns or dynamics can be identified.

**NSR:** Chinese scientists have made important contributions to our understanding of critical issues related to mass extinctions in Earth history. Could you identify some of their contributions, and provide some suggestions for their future research?

**DHE:** Chinese geologists have been at the leading edge of work on mass extinctions for decades. In the mid-1980s most of the useful work on the end-Permian mass extinction was done by Chinese paleontologists, particularly at the Nanjing Institute of Geology and Paleontology and China University of Geosciences in Wuhan, reflecting the presence of a majority of good marine sections across the Permian -Triassic boundary in China. As mass extinctions became a major interest of geologists around the world, new generations of paleontologists and other geologists were drawn into these questions. Today, the study of mass extinctions now requires expertise in many different techniques, from statistics and phylogenetic analysis to

Chinese geologists have been at the leading edge of work on mass extinctions for decades.

—Douglas H. Erwin

carbon, oxygen isotopes and elemental geochemistry and high-resolution geochronology. In addition, high-resolution global biodiversity patterns produced with artificial intelligence approaches and high-performance computing technologies will provide many more details about mass and minor extinctions (e.g. [7]). Groups in China have led many international collaborations to address these problems.

Southern China has by far the best marine record of the end-Permian mass extinction in the world. There may be as many Permian-Triassic boundary sites in China as in the rest of the world combined, and some of these sections have been studied for many decades. The Global Boundary Stratotype Section and Point (GSSP), the global reference for the Permian-Triassic boundary is located at the Meishan site between Nanjing and Shanghai (along with other GSSPs). Groups at Nanjing University, the Nanjing Institute of Geology and Palaeontology and at China University of Geosciences in Wuhan have published hundreds of important papers on various aspects of the marine extinction. The frequent volcanism near the South China block during the late Permian produced many volcanic ash beds interbedded with the fossiliferous deposits, and since the mid-1990s the Nanjing group has been working with geochronologists at MIT to develop a very high-resolution timescale for this event, allowing many proposed causes to be evaluated.

Beyond the EPME, the end-Guadalupian mass extinction and its possible relationship to the Emeishan large igneous province have long attracted attention from several different groups, as has the end-Ordovician mass extinction, with several well-preserved sections in China.

**Figure 2:**
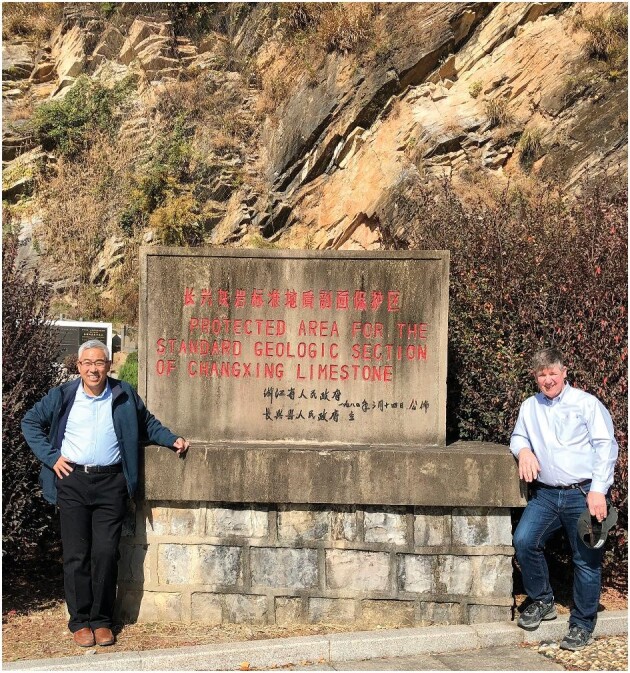
Profs. Douglas H. Erwin and Shuzhong Shen at the Meishan Section D in Changxing, Zhejiang Province in 2019. The Meishan sections have been extensively studied by the US-Sino joint research team on the end-Permian mass extinction. The monument was established on the 14th of March, 1980 and behind is the Changhsing Limestone of the Meishan Section D with two GSSPs. *(Courtesy of Shuzhong Shen)*

**NSR:** Despite these biotic crises, life has persisted. So how do ecosystems start to recover after a crisis? Are there any early warnings of tipping points, or coming instability of the complex Earth system, and what could geological history tell us about the exploitation and protection of modern ecosystems?

**DHE and SZS:** These issues of resilience and recovery are among the most important currently under study by paleobiologists, geochemists and evolutionary biologists. Indeed, questions about resilience are of broad interest to ecologists and other biologists, economists, physicists, and those concerned with policy. While there is no guarantee that research on past biotic crises will be relevant in these other areas, as paleontologists we do have an incredible record of natural experiments in which systems have been perturbed and responded to those perturbations. Properly studied, these events can provide examples to understand the impact of environmental perturbations on ecological communities, what elements of a community persist, and how communities reform after a perturbation. By comparing smaller biotic crises to mass extinctions, paleontologists can search for common patterns. Ideally, we can then use this knowledge to build models, including quantitative models, of such events and then compare insights from our work with ecologists or economists to search for a more general understanding of resilience and recovery.

Focusing again on the end-Permian mass extinction (252 Ma), two decades ago we thought that there was a lag of perhaps 5 million years before life really began to recover from this crisis. Research over the past decade has shown that the recovery was much more rapid. For example, recent work by a group from China University of Geosciences, Wuhan described an extraordinary fauna from Guiyang dating only 1 million years after the extinction including fish, mollusks, and arthropods [8]. So, despite the catastrophic loss of perhaps 90% of all marine species and many terrestrial plants and animals, diverse early Triassic ecosystems are constructed almost as rapidly as the recovery after the end-Cretaceous mass extinction.

The increasingly widespread adoption of high-resolution geochronology to illuminate the pace of mass extinctions and subsequent recoveries has demonstrated that both were far more rapid than expected: global collapses occurred in just tens of thousands of years, and recoveries were almost as rapid. Such results may provide important lessons for our current biodiversity crisis. But it is important to remember the foundational research that underpins these conclusions: intensive fieldwork and collection of fossils and samples for sedimentological, geochemical and geochronological analysis, precise local, regional and global correlations of geological sections, detailed studies of fossils and laboratory analysis of samples, big data and high-performance computing technology, all of which feed into an integrated view of these events which allows us to test various hypotheses.

## Author Biography

Shucheng Xie is a professor from State Key Laboratory of Biogeology and Environmental Geology, China University of Geosciences at Wuhan, Editorial Board member of NSR, and Guest Editor of the Special Topic.
